# Isoforms of soluble vascular endothelial growth factor in stress-related mental disorders: a cross-sectional study

**DOI:** 10.1038/s41598-021-96313-8

**Published:** 2021-08-17

**Authors:** Johanna Wallensten, Fariborz Mobarrez, Marie Åsberg, Kristian Borg, Aniella Beser, Alexander Wilczek, Anna Nager

**Affiliations:** 1 Academic Primary Health Care Centre, Region Stockholm, Solnavägen 1E, Box 45436, 104 31 Stockholm, Sweden; 2 Department of Clinical Sciences, Karolinska Institutet, Danderyd University Hospital, 18288 Stockholm, Sweden; 3 Department of Medical Sciences, Uppsala University, 75185 Uppsala, Sweden; 4 Division of Family Medicine and Primary Health Care, Department of Neurobiology, Care Sciences and Society, Karolinska Institutet, 17177 Stockholm, Sweden

**Keywords:** Depression, Stress and resilience

## Abstract

Vascular endothelial growth factor (VEGF) has been implicated in the pathophysiology of stress-related mental disorders. However, VEGF levels have seldom been compared across mental disorders and never by isoforms. Pathophysiological processes involving leakage of astrocyte-derived extracellular vesicles (EVs) across the blood–brain barrier could be associated with VEGF levels in patients with stress-related mental disorders. This cross-sectional study compared plasma levels of VEGF_121_, VEGF_165_, and VEGF_121_ + VEGF_165_ (VEGF_total_) in patients with stress-induced exhaustion disorder (SED) (n = 31), patients with major depressive disorder (MDD) (n = 31), and healthy controls (n = 61). It also analyzed the correlation between VEGF and astrocyte-derived EVs in plasma. An enzyme-linked immunosorbent assay (ELISA) was used to measure VEGF_121_ and VEGF_165_ in citrate plasma, and flow cytometry was used to measure astrocyte-derived EVs in plasma. The mean concentration of soluble VEGF_121_ (sVEGF_121_) was significantly higher in patients with SED than healthy controls (*P* = 0.043). Mean sVEGF_165_ was significantly lower in patients with MDD than patients with SED (*P* = 0.004) or healthy controls (*P* = 0.037). Mean sVEGF_total_ was significantly higher in patients with SED than in patients with MDD (*P* = 0.021) and also higher in patients with SED than healthy controls (*P* = 0.040). Levels of sVEGF_121_ were positively correlated with levels of astrocyte-derived EVs only in patients with SED (*P* = 0.0128). The same was true of levels of sVEGF_total_ and astrocyte-derived EVs (*P* = 0.0046). Differing levels of VEGF isoforms may reflect different pathophysiological mechanisms in SED and MDD. Further research is needed to better understand the potential roles of VEGF isoforms and astrocyte-derived EVs in mental disorders.

## Introduction

Acute and chronic stress are important in the development of many mental disorders, such as posttraumatic stress disorder, schizophrenia, and major depressive disorder^1^. The pathophysiological pathways between stress and these disorders remain unclear^2,3^, but research suggests that cerebrovascular and endothelial dysfunction may play a role^4^. Vascular endothelial growth factor (VEGF) is one potential component of the pathways. VEGF is important in angiogenesis and blood vessel formation, has neurotrophic and neuroprotective effects^5^, and promotes blood–brain barrier permeability^6,7^. Research shows that it is involved in the pathophysiology of major depressive disorder (MDD)^8^ and in the effects of antidepressant treatment^9–11^.

Previous studies have examined VEGF concentrations in the peripheral blood of patients with MDD^8,12–14^ and stress-induced exhaustion disorder (SED)^15–18^. SED is a clinical condition defined by at least 6 months of chronic stress without sufficient recovery^19^. It has been classified as a disorder in the Swedish version of the ICD-10 since 2004. In other countries, SED is sometimes classified as a kind of depression (i.e. job stress-induced depression)^20^ or may be referred to as clinical burnout^21^ or chronic burnout syndrome^22^.

Previous studies of VEGF in patients with SED^15–18^ and MDD^12–14^ have produced conflicting results, which might be caused, at least in part, by differing study designs. For instance, studies may have measured different isoforms of the VEGF family. VEGF_121_ and VEGF_165_ are the two major isoforms in mammals^23^. VEGF_121_, the main isoform in circulating blood, probably plays a minor role in angiogenesis but a major role in vascular permeability^24^. The heavier isoform, VEGF_165_, has higher mitogenic potential and appears to induce angiogenesis^25–28^. Previous research has investigated plasma levels of VEGF in people with different mental disorders^14^, but to the best of our knowledge, no previous research has compared plasma concentrations of different isoforms of VEGF in patients with different mental disorders.

Additionally, research indicates that VEGF mediates increased permeability of the blood–brain barrier (BBB)^29–32^. Particles that may indicate increased BBB permeability have been identified in the peripheral blood of patients with SED, and to a lesser extent, MDD^33^. These particles, astrocyte-derived extracellular vesicles (EVs), are important in intercellular communication and are released during cellular activation or death^34,35^. They include both smaller exosomes and larger microvesicles (sometimes called microparticles)^35^. Because the presence of elevated levels of EVs in peripheral blood may be related to stress and BBB permeability^33^, we hypothesized that levels of VEGF in plasma would be correlated with levels of EVs in plasma.

To better understand the role of VEGF in stress-related mental disorders, the present study aimed to compare plasma levels of different isoforms of VEGF, including VEGF_121_, VEGF_165_, and VEGF_121_ + VEGF_165_ (VEGF_total_) in patients with SED, patients with MDD, and healthy controls. We also analyzed the correlation between levels of VEGF and astrocyte-derived EVs in plasma.

## Materials and methods

### Study population

Between 2014 and 2018, patients with common mental disorders treated at the psychiatric outpatient clinic at Ersta Hospital, Stockholm, were consecutively recruited to the study. During 2018, patients with MDD attending an outpatient clinic in Stockholm, the Capio Anxiety and Depression Clinic, were also consecutively recruited. Recruitment continued until the SED and MDD groups reached predefined sizes calculated on the basis of a pilot study.

Patients who fulfilled the diagnostic criteria for SED^19^ (Table 1) or MDD (DSM-5) were asked to participate in the study by their physician, occupational therapist, or nurse. Inclusion criteria were ongoing SED or MDD diagnosed less than 3 months prior to inclusion, age 18–65 years, the ability to understand Swedish, and the capacity to undergo 30–40 min of clinical examination. Patients with SED could also fulfill criteria for depression if the physician considered the depressive symptoms secondary to SED. Exclusion criteria were anemia, vitamin B12 deficiency, subclinical thyroid disease, alcohol overconsumption, and a somatic (e.g. anemia) or psychiatric (e.g. post-traumatic stress disorder) diagnosis that better explained the patient’s symptoms.

**Table 1:** Criteria for stress-induced exhaustion disorder according to the Swedish National Board of Health and Welfare and Swedish version of the International Classification of Diseases, 10th edition (code F-43.8).

A. Physical and mental symptoms of exhaustion for at least 2 weeks. The symptoms have developed in response to one or more identifiable stressors present for at least 6 months
B. The clinical picture is dominated by markedly reduced mental energy, as manifested by reduced initiative, lack of endurance, or increased time needed for recovery after mental effort
**C. At least four of the following symptoms have been present, nearly every day, during the same 2-week period**
Concentration difficulties or impaired memory
Markedly reduced capacity to tolerate demands or to work under time pressure
Emotional instability or irritability
Sleep disturbance
Marked fatigability or physical weakness
Physical symptoms such as aches and pains, palpitations, gastrointestinal problems, vertigo, or increased sensitivity to sound
D. The symptoms cause clinically significant distress or impairment in occupational, social, or other important respects
E. The symptoms are not due to the direct physiological effects of a substance (e.g. a drug of abuse, a medication) or a physical illness/injury (e.g. hypothyroidism, diabetes, infectious disease)

Criteria A through E must be fulfilled to diagnose stress-induced exhaustion disorder.

Physician examination and blood analyses were used to check for inclusion and exclusion criteria. Patients were diagnosed with SED or MDD by their physician. To exclude other psychiatric diagnoses, patients underwent the Swedish version of the Mini International Neuropsychiatic Interview (M.I.N.I.) 6.0.0^36,37^. M.I.N.I. was administered by a member of the research staff, a clinic psychologist, or a physician, all of whom were familiar with the instrument. Clinical characteristics such as height and weight were also gathered, as was information on use of antidepressant medication. 24 patients with MDD, 25 patients with SED, and no healthy controls had antidepressant medication.

Controls, matched as closely as possible for age and sex, were chosen from a group of 165 healthy subjects described in detail in a previous publication^17^. In brief, the healthy subjects were recruited by Statistics Sweden, Sweden’s national statistics agency, in 2009. Inclusion criteria were being between the ages of 28 and 55 and being a permanent resident of the Stockholm area. The exclusion criteria were current or previous physical and/or mental disorders. Physician examination, including with the Structured Clinical Interview for Mental Disorders (SCID)^38^, and blood samples was used to check for inclusion and exclusion criteria. The healthy controls provided blood samples.

### Symptom rating scales

Two self-rated scales were used to measure depressive and cognitive symptoms. Severity of depressive symptoms was assessed with the 9-item self-reported version of the Montgomery-Asberg Depression Rating Scale (MADRS-S). Higher scores on the scale reflect more severe symptoms^39,40^. The 25-item Cognitive Failures Questionnaire (CFQ) was used to quantify cognitive problems. The CFQ measures self-reported cognitive failures in daily life. High scores indicate a high degree of subjective cognitive problems^41,42^. Patients completed the CFQ; people in the control group did not.

### Sample collection

Blood samples were obtained from healthy controls in 2009 in accordance with a standardized protocol presented elsewhere^33^. In summary, all participants were asked to abstain from consuming alcohol prior to their blood test. They were also asked to fast from midnight on the previous night, to avoid physical activity prior to blood sampling, and to put off sampling if they had any symptoms of infection. Blood samples were drawn in the morning after the participant had rested for at least 15 min. The samples were drawn into citrated tubes through direct venepuncture from an antecubital vein using a 21G sampling needle. They were centrifuged within 1 h at 2000*g* for 20 min at room temperature and subsequently stored at − 80 °C as platelet poor plasma. Blood samples were obtained from patients at inclusion using the same procedure as for controls. Samples from patients and controls were analyzed in the same batch at Danderyd University Hospital, Sweden.

### Measurement of sVEGF_121_ and sVEGF_165_

Soluble(s) VEGF_121_ and sVEGF_165_ were measured with an enzyme-linked immunosorbent assay (ELISA) following the manufacturer’s recommendations (LSBio, Seattle, WA, USA). Citrate plasma was thawed in a water bath for approximately 5 min at 37 °C. Standards and samples were added in pre-coated wells with either sVEGF_121_ or sVEGF_165_. After incubation (1 h at 37 °C) and wash, the detection antibody was added. After a second incubation (1 h at 37 °C) and a second wash, conjugate was added, and the plates were incubated for 30 min at 37 °C. The plates were washed, Tetramethylbenzidine substrate was added, and after 20 min of incubation (37 °C), 50 µl of stop solution was added to each well. Optical density was read using a microplate reader set to 450 nm with the correction set to 570 nm. Intra-assay and inter-assay variation were less than 15% for both assays. Data are presented as picograms (pg)/ml sVEGF_121,_ sVEGF_165_, and sVEGF_total_ (VEGF_121_ plus VEGF_165_).

### Analysis of astrocyte-derived extracellular vesicles

A detailed description of the analysis of EVs has been presented elsewhere^33^. In summary, EVs were measured in platelet poor plasma collected as described above. After thawing, 20 µl of samples were incubated with markers specific to the astrocytes aquaporin-4 (AQP4) and glial fibrillary acidic protein (GFAP). These markers were anti-Aquaporin-4 Dylight 488 (corresponding to Human Aquaporin 4 aa 50–150, Abcam, Cambridge, UK), and/or anti-GFAP Dylight 755 (Abcam, Cambridge, UK). The samples were measured on a Beckman Gallios instrument (Beckman coulter, Brea, CA, USA), a flow cytometer with the threshold set to forward scatter.

Astrocyte-derived EVs were defined by size (forward/side scatter characteristics, ≤ 0.9 µm) and expression of AQP4 and GFAP. EVs were grouped into three subgroups: single AQP4 expression, single GFAP expression, and double expression (AQP4 and GFAP) Results are presented as EVs/µl plasma. Both AQP-4 and GFAP are predominately expressed astrocytes^43^ but can also be found in detected in smaller quantities elsewhere^44–46^. Thus, we chose to include double-positive EVs (i.e. AQP4-positive and GFAP-positive) in the analysis because we could be more confident that they derived from astrocytes. Results are presented as EVs/µl plasma.

### Statistical methods

**Figure 1:**
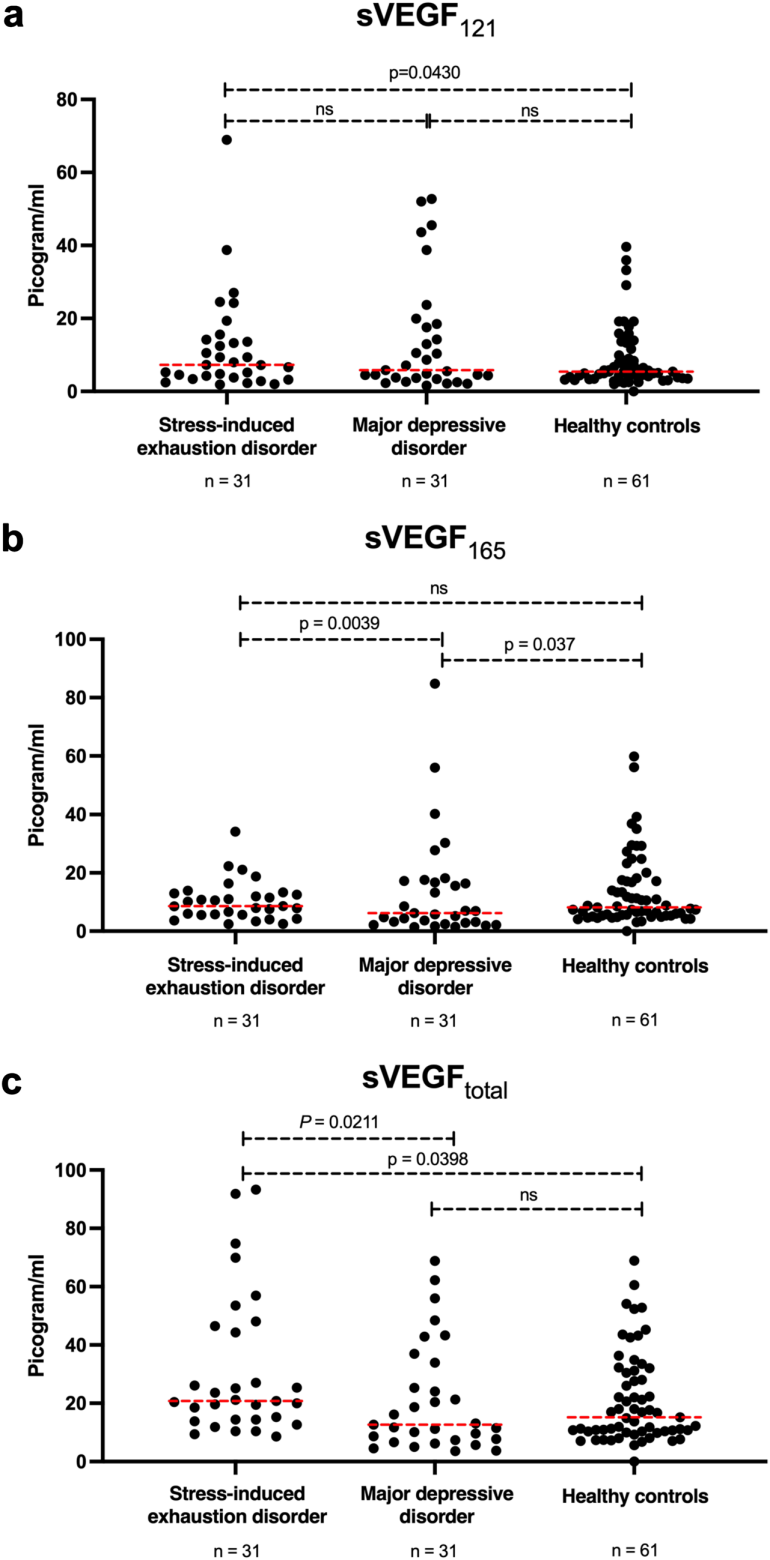
Plasma levels of vascular endothelial growth factor (VEGF)_121_ and VEGF_165_ as measured by enzyme-linked immunosorbent assay (ELISA). Data are presented as VEGF_121_, VEGF_165_, and VEGF_total_ (VEGF_121_ plus VEGF_165_); *ns* not significant.

Clinical and demographic characteristics were compared between groups, as appropriate, with non-parametric or parametric tests. The Kruskal–Wallis test was used for non-parametric tests of three groups and the Mann–Whitney test for two groups. ANOVA was used for parametric tests of three groups and un-paired t-tests for parametric tests of two groups. Bonferroni was used as a post-hoc test of Kruskal–Wallis and ANOVA tests (Table 2). Plasma levels of sVEGF isoforms (sVEGF_121,_ sVEGF_165_, and sVEGF_total_) were compared between groups with ANOVA (after log-transformation) together with Bonferroni post-hoc test (Fig. 1). Correlations between EV subtypes (AQP4-positive, GFAP-positive, and double-positive) and sVEGF isoforms (sVEGF_121,_ sVEGF_165_, and sVEGF_total_) were investigated with linear regression after log-transformation (Table 3). Correlations between symptom rating scales and sVEGF and EVs were investigated by Pearson Correlation tests. *P* values of ≤ 0.05 were considered significant. Statistical analysis was performed with SPSS Statistics (IBM SPSS Statistics for Windows, v 26.0. Armonk, NY: IBM Corp.) and JMP software (SAS Institute, v12.0, Cary, North Carolina, USA).

**Table 2:** Clinical and demographic characteristics of patients with stress-induced exhaustion disorder (n = 31), patients with major depressive disorder (n = 31), and healthy controls (n = 61).

Clinical and demographic characteristics	Stress-induced exhaustion disorder	Major depressive disorder	Healthy controls	*P* value
Mean age in years	n = 31 44.6 (9.7)	n = 31 40.3 (10·8)	n = 61 42.2 (9.5)	0.206
Women	n = 31 27 (87.1%)	n = 31 26 (83.9%)	n = 61 52 (85.2%)	0.999
Mean BMI	n = 31 24.8 (5.5)	n = 31 25.0 (5.0)	n = 61 24.7 (3.6)	0.898
Mean MADRS-S sum	n = 22 19.9 (5.6)	n = 25 27.1 (7.9)	n = 61 5.0 (3.6)	0.001*
Mean CFQ sum	n = 14 57.7 (11.0)	n = 26 50.0 (12.1)	n = 0	0.025

Data are mean (SD) or n (%).

*BMI* body mass index, *MADRS-S* self-reported version of the Montgomery-Asberg Depression Rating Scale, *CFQ* Cognitive Failures Questionnaire.

*Significant difference between patients with stress-induced exhaustion disorder and healthy controls and between patients with major depressive disorder and healthy controls.

**Table 3:** Correlation between levels of vascular endothelial growth factor (VEGF) isoforms and astrocyte-derived extracellular vesicles, including aquaporin-4 (AQP4), glial fibrillary acidic protein (GFAP), and AQP4 + GFAP in patients with stress-induced exhaustion disorder, patients with major depressive disorder, and healthy controls.

	AQP4	GFAP	AQP4 + GFAP
**Stress-induced exhaustion disorder (n = 31)**			
VEGF_121_			
* P*	Non-significant	Non-significant	0.0128
* r*^2^	No correlation	No correlation	0.19
VEGF_165_			
* P*	Non-significant	Non-significant	Non-significant
* r*^2^	No correlation	No correlation	No correlation
VEGF_total_			
* P*	Non-significant	Non-significant	0.0046
* r*^2^	No correlation	No correlation	0.25
**Major depressive disorder (n = 31)**			
VEGF_121_			
* P*	Non-significant	Non-significant	Non-significant
* r*^2^	No correlation	No correlation	No correlation
VEGF_165_			
* P*	Non-significant	Non-significant	Non-significant
* r*^2^	No correlation	No correlation	No correlation
VEGF_total_			
* P*	Non-significant	Non-significant	Non-significant
* r*^2^	No correlation	No correlation	No correlation
**Healthy controls (n = 61)**			
VEGF_121_			
* P*	Non-significant	Non-significant	Non-significant
* r*^2^	No correlation	No correlation	No correlation
VEGF_165_			
* P*	Non-significant	Non-significant	Non-significant
* r*^2^	No correlation	No correlation	No correlation
VEGF_total_			
* P*	Non-significant	Non-significant	Non-significant
* r*^2^	No correlation	No correlation	No correlation

### Ethics

The study was approved by the Regional Ethical Review Board in Stockholm, Sweden, http://www.epn.se/en/start/, d.nr. 2008/0061-31, 2014/585-31/1, 2016/1239-32, 2017/2088-32. It was carried out in accordance with the recommendations of the Local Ethics Committee, Karolinska Institutet, Stockholm, and the Declaration of Helsinki. All participants received verbal and written information about the study and provided written informed consent prior to participation. Data were pseudonymized before they were linked and analyzed.

## Results

### Clinical and demographic characteristics

The study included 31 patients with SED, 31 patients with MDD, and 61 healthy controls. The three groups were similar in age, sex, and body mass index (Table 2). Differing mean MADRS-S scores reflected the participants’ diagnoses or lack thereof. They were highest in patients with MDD, second highest in patients with SED, and lowest in healthy controls (*P* = 0.001). Higher mean CFQ scores were observed in patients with SED than in patients with MDD (*P* = 0.025).

In those with SED, there was no significant difference in plasma levels of sVEGF_121_, sVEGF_165,_ or sVEGF_total_ in patients who received and who did not receive antidepressants (*P* = 0.385 for sVEGF_121,_ *P* = 0.957 for sVEGF_165_ and *P* = 0.746 for sVEGF_total_). In addition, there were no significant differences in concentration of leucocytes, erythrocytes, or platelets between patients with SED who received antidepressant medication and patients with SED who did not receive such medication.

### Isoforms of VEGF

There were statistically significant differences between the groups (Fig. 1). The mean plasma concentration of sVEGF_121_ was significantly higher in patients with SED (15.4, SD ± 1.9 pg/ml) than in healthy controls (8.7, SD ± 1.4 pg/ml), *P* = 0.043. Mean sVEGF_165_ was significantly lower in patients with MDD (9.5, SD ± 1.8 pg/ml) than in patients with SED (15.9, SD ± 1.8 pg/ml, *P* = 0.004) or healthy controls (12.3, SD ± 1.2 pg/ml, *P* = 0.037). The largest differences between the groups were observed in plasma levels of sVEGF_total_. These levels were significantly higher in patients with SED (31.2, SD ± 3.3 pg/ml) than in patients with MDD (21.1, SD ± 3.4 pg/ml, *P* = 0.021) and also higher in patients with SED than in healthy controls (21.1, SD ± 2.4 pg/ml, *P* = 0.040). There was a significant positive correlation between MADRS-S scores and levels of AQP4 (*r* = 0.196 *P* = 0.042) and between MADRS-S scores and levels of GFAP (*r* = 0.210 *P* = 0.029). There was no significant correlation between MADRS-S scores and levels of VEGF_total_ or between scores on the CFQ symptom rating scale and VEGF isoforms or EVs.

### Correlation between sVEGF isoforms and astrocyte-derived extracellular vesicles

In patients with SED, there was a significant positive correlation between the plasma concentration of sVEGF_121_ and astrocyte-derived EVs concurrently expressing AQP4 and GFAP (*P* = 0.0128), as well as between sVEGF_total_ and astrocyte-derived EVs concurrently expressing these markers (*P* = 0.0046) (Table 3).

## Discussion

In the present study, we demonstrated that patients with SED had sVEGF_121_ levels that were significantly higher than the levels in healthy controls. Patients with MDD had significantly lower levels of sVEGF_165_ than either patients with SED or healthy controls. Additionally, we observed that levels of sVEGF_121_ and of sVEGF_total_ were positively correlated with plasma levels of astrocyte-derived EVs in patients with SED but not in patients with MDD or healthy controls.

It is challenging to compare our findings with those of previous studies on the connection between VEGF and either SED or MDD because in prior work, results have not been reported by VEGF isoform. The results of previous studies of VEGF (isoforms not reported) in people with SED differ. Some researchers have found higher levels of VEGF in people with SED than healthy controls^17,18^, others have found similar levels^15^, and still others have found lower levels^16^. Findings of elevated levels of VEGF might be related to measuring VEGF_121_, and findings of no difference, to measuring VEGF_165._ Different isoforms are thus a potential cause of variation in plasma levels of VEGF across studies.

Differences in methodology probably contributed to the heterogeneity in study findings. Choice of protein assay^15^ can lead to significantly different results, as can the decision to use ELISA methods or multiplex assays^47^ and the decision to measure VEGF in plasma or serum^48^. Measuring circulating extracellular VEGF in plasma is often more accurate than measuring it in serum^49^ because peripheral VEGF can be stored in blood cells such as platelets and released during the clotting process. Centrifugation time and speed may also affect VEGF concentrations^50^.

Our finding of significantly lower levels of sVEGF_165_ in patients with MDD than in healthy controls contradicts the findings of two previous meta-analyses, which found that levels of sVEGF (isoforms not reported) were higher in the peripheral blood of patients with MDD than in the peripheral blood of healthy controls^12,13^. However, a 2020 meta-analysis^14^ that had a larger sample size (> 4000 participants; isoforms not reported) examined the levels of VEGF in people with different psychiatric disorders, including MDD, and healthy controls. The analysis showed significant elevation of blood levels of VEGF, but only in patients with MDD who were treated with antidepressants. Levels of VEGF may also vary across the course of a disorder^17^, which would mean that the timing of measurement is important, and inclusion and exclusion criteria could be crucial.

Different isoforms, disease severity, and antidepressant treatment could all play a role in the inconsistent findings regarding VEGF in people with mental disorders, and it is still unclear whether changes in VEGF levels are part of the causal pathway in depression, a result of depression^13^, or both.

If confirmed in other studies, our finding of elevated levels of VEGF_121_ in the peripheral blood of patients with SED may help illuminate physiological changes associated with the disorder. Previous research from our group suggests that patients with SED may have increased BBB permeability^33^. In that study, we found raised levels of astrocyte-derived EVs in the peripheral blood of patients with SED. Those findings were consistent with other researchers’ findings of leakage or release of astrocyte-derived EVs through the blood brain barrier in patients with traumatic brain injury^43,51^. The sVEGF_121_ isoform is the one most closely associated with increased vascular permeability^52^. Thus, raised levels of sVEGF_121_ in the peripheral blood of patients with SED could help explain increased BBB permeability in these patients.

In the current study, we also found that levels of sVEGF_121_ were correlated with levels of circulating astrocyte-derived EVs in patients with SED. This finding is consistent with the hypothesis that sVEGF_121_ is involved in the physiological changes that result in increased levels of astrocyte-derived EVs in the peripheral blood of patients with SED.

### Limitations

This study has several limitations. First, blood samples were obtained from patients and healthy controls at different times, and differences in storage times could have affected the results. However, the blood sampling routines were the same in both groups, and the samples were analyzed in the same batches. Second, patients with SED and MDD may have been in different stages of their diseases. To minimize this diversity, all patients included in the study, regardless of diagnosis, had to be diagnosed less than three months before blood sampling. Third, the cross-sectional design makes it impossible to draw causal inferences on the basis of the observed associations.

## Conclusions

Our study indicates that plasma levels of VEGF isoforms vary in patients with SED, patients with MDD, and healthy controls. These heterogeneous levels may reflect different pathophysiological mechanisms in SED and MDD. Further research is needed to better understand the potential roles of isoforms of VEGF in mental disorders, including whether stress can influence processes involving VEGF and BBB permeability in people with stress-related mental disorders such as SED and MDD.

## Data Availability

The dataset generated and analyzed during the study is available from the corresponding author on reasonable request.
