# Author Correction: Isoforms of soluble vascular endothelial growth factor in stress-related mental disorders: a cross-sectional study

**DOI:** 10.1038/s41598-023-37259-x

**Published:** 2023-06-23

**Authors:** Johanna Wallensten, Fariborz Mobarrez, Marie Åsberg, Kristian Borg, Aniella Beser, Alexander Wilczek, Anna Nager

**Affiliations:** 1 Academic Primary Health Care Centre, Region Stockholm, Solnavägen 1E, Box 45436, 104 31 Stockholm, Sweden; 2 Department of Clinical Sciences, Karolinska Institutet, Danderyd University Hospital, 18288 Stockholm, Sweden; 3 Department of Medical Sciences, Uppsala University, 75185 Uppsala, Sweden; 4 Division of Family Medicine and Primary Health Care, Department of Neurobiology, Care Sciences and Society, Karolinska Institutet, 17177 Stockholm, Sweden

Correction to: *Scientific Reports* 10.1038/s41598-021-96313-8, published online 17 August 2021

The original version of this Article contained errors in the Materials and methods as well as the Discussion sections.

In the Materials and methods section, under the subheading ‘Study population’,

“Between 2016 and 2018, patients with common mental disorders treated at the psychiatric outpatient clinic at Ersta Hospital, Stockholm, were consecutively recruited to the study.”

now reads:

“Between 2014 and 2018, patients with common mental disorders treated at the psychiatric outpatient clinic at Ersta Hospital, Stockholm, were consecutively recruited to the study.”

And,

“All patients with MDD, five patients with SED, and no healthy controls had antidepressant medication.”

now reads:

“24 patients with MDD, 25 patients with SED, and no healthy controls had antidepressant medication.”

Additionally, under the subheading ‘Ethics’,

“The study was approved by the Regional Ethical Review Board in Stockholm, Sweden, http://www.epn.se/en/start/, d.nr. 2014/585-31/1, 2016/1239-32 and 2017/970-21/1.”

now reads:

“The study was approved by the Regional Ethical Review Board in Stockholm, Sweden, http://www.epn.se/en/start/, d.nr. 2008/0061-31, 2014/585-31/1, 2016/1239-32, 2017/2088-32.”

In the Discussion section, under the subheading ‘Limitations’,

“Third, the effect of antidepressant medication on plasma levels of VEGF in different diagnostic groups could not be clarified, since all patients with MDD, only five patients with SED, and none of the healthy controls were treated with antidepressants. Thus, a possible impact of antidepressant medication on the results cannot be eliminated. Fourth, the cross-sectional design makes it impossible to draw causal inferences on the basis of the observed associations.”

now reads:

“Third, the cross-sectional design makes it impossible to draw causal inferences on the basis of the observed associations.”

The original Article has been corrected.

